# Association between four nontraditional lipids and ischemic stroke: a cohort study in Shanghai, China

**DOI:** 10.1186/s12944-022-01683-1

**Published:** 2022-08-16

**Authors:** Minhua Tang, Qi Zhao, Kangqi Yi, Yiling Wu, Yu Xiang, Shuheng Cui, Xuyan Su, Yuting Yu, Genming Zhao, Yonggen Jiang

**Affiliations:** 1 Department of Epidemiology, School of Public Health, Key Laboratory of Public Health Safety of Ministry of Education, Fudan University, Shanghai, 200032 China; 2 Songjiang District Center for Disease Control and Prevention, Shanghai, 201600 China

**Keywords:** Nontraditional lipids, Ischemic stroke, Cohort study

## Abstract

**Background:**

The correlation between nontraditional lipids and ischemic stroke (IS) is inconsistent and controversial. This study aimed to examine the association of four nontraditional lipids with IS risk in Chinese adults.

**Methods:**

This prospective community-based cohort study was performed in Songjiang District, Shanghai, China. The study began in 2016 and included 34,294 participants without stroke before the investigation. The association between nontraditional lipids (nonhigh-density lipoprotein cholesterol [non-HDL-C], total cholesterol/high-density lipoprotein cholesterol [TC/HDL-C], triglyceride [TG]/HDL-C, and low-density lipoprotein cholesterol [LDL-C]/HDL-C) and IS was studied with multivariate Cox regression models. The dose–response associations between these four serum lipids and IS were explored using restricted cubic spline (RCS) analysis.

**Results:**

There were a total of 458 IS cases with 166,380 person-years of follow-up. Compared with the lowest tertiles, the highest tertiles of the nontraditional blood lipids showed greater IS risk after controlling for potential confounders. The hazard ratios (*HRs*) and 95% confidence intervals (95% *CIs*) were as follows: TC/HDL-C, 1.63 (1.28–2.07); TG/HDL-C, 1.65 (1.28–2.13); LDL-C/HDL-C, 1.51 (1.18–1.92); and non-HDL-C, 1.43 (1.13–1.81). The fully adjusted RCS curves presented a nonlinear relationship, and the risk increased when the TC/HDL-C, TG/HDL-C, and LDL-C/HDL-C levels were > 3.47, > 0.92, and > 1.98, respectively.

**Conclusions:**

This community-based cohort study presents a positive association between the four nontraditional lipids and IS incidence. Maintaining relatively low lipid ratios can be beneficial for preventing stroke. Nontraditional lipids can be considered targets for managing blood lipids.

**Supplementary Information:**

The online version contains supplementary material available at 10.1186/s12944-022-01683-1.

## Introduction

Stroke, a vascular disease that causes acute focal lesions in the central nervous system [1], is characterized by high morbidity, recurrence, disability, mortality, and high economic burden [2]. Stroke has become the primary reason for disability and mortality among Chinese adults [3]. From 1990 to 2019, the absolute number of global stroke events increased by 70%, and the number of stroke-related deaths increased by 43% [4]. Ischemic stroke (IS) is the most common stroke type, and its incidence continues to rise in China [2].

Dyslipidemia plays an essential role in the development of stroke [5, 6]. It has been proposed that lowering low-density lipoprotein cholesterol (LDL-C) is a major indicator for preventing and controlling atherosclerotic cardiovascular disease (ASCVD) in lipid management [7, 8]. However, even when LDL-C is within normal limits, cardiovascular events occur due to increased serum triglycerides (TGs) or decreased high-density lipoprotein cholesterol (HDL-C) concentrations, a condition known as residual cardiovascular risk [9, 10]. Therefore, exploring the impact of novel lipid markers on cardiovascular disease (CVD) has become a hot topic in cardiovascular research.

More recently, the evidence supported the idea that nontraditional lipids such as non-HDL-C, total cholesterol (TC)/HDL-C, TG/HDL-C, and LDL-C/HDL-C played a significant role in stroke events and indicated that they were more robust predictors than traditional lipids [11–13]. However, some researchers reported inconsistent results. For example, a cohort study in Paris observed that lipid ratios were not significantly associated with IS [14]. Therefore, the role of nontraditional lipids in stroke requires further study. Moreover, research on associations between these lipids and IS in Shanghai, China, is scarce, and few studies have reported dose–response relationships to explore the risk threshold. Lipids are an easily accessible modifiable cardiovascular risk factor, and the aforementioned nontraditional lipids are easy to calculate [15]. Serum lipids are associated not only with stroke but also with obesity and diabetes, which are all factors related to adverse cardiovascular events [16, 17].

Therefore, to better discuss the problems above, the study investigated the relations between four nontraditional blood lipids and IS outcomes among populations in Shanghai communities based on a cohort study.

## Methods and materials

### Study design

**Fig. 1:**
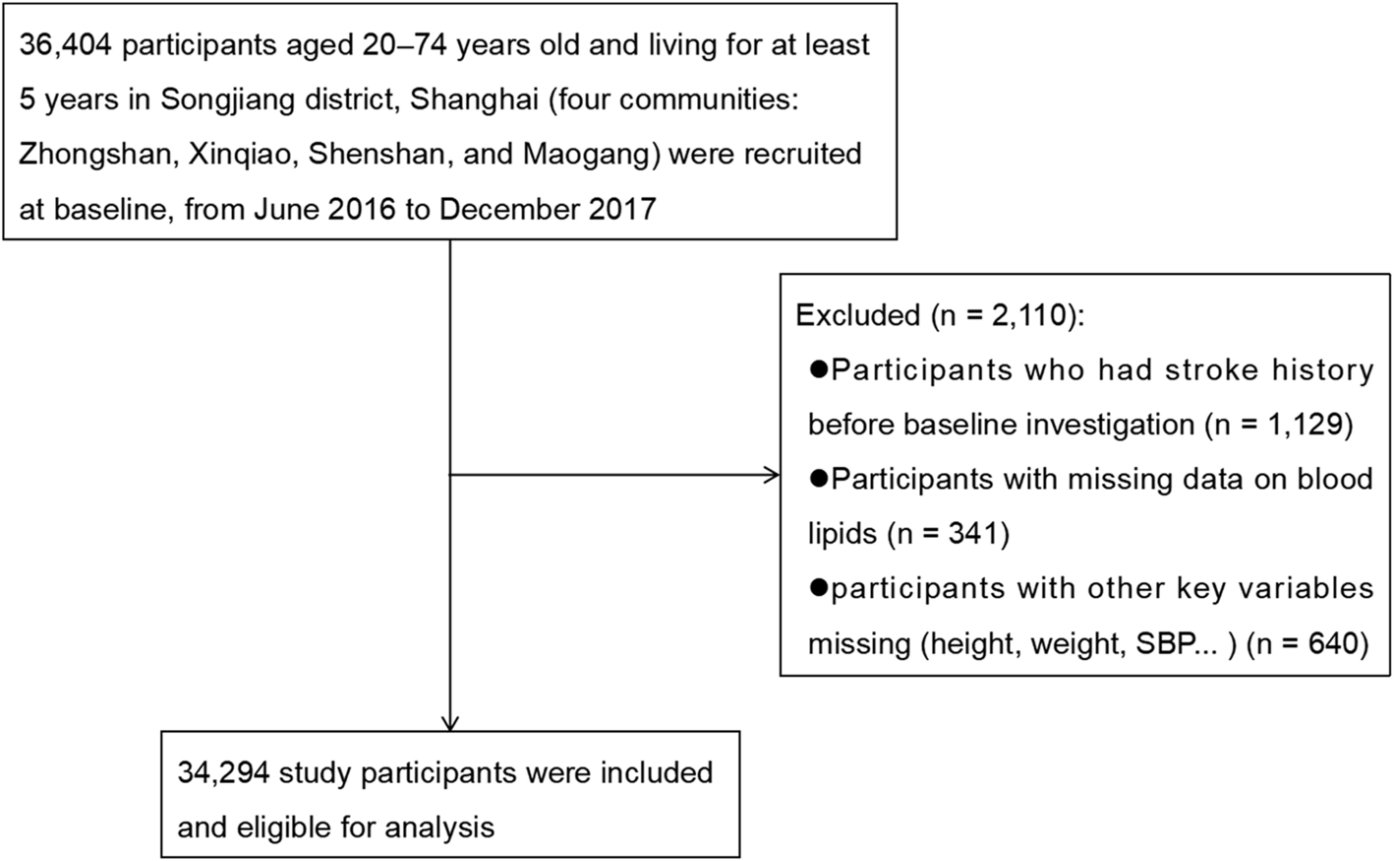
Selection process of the participants

The present data were obtained from the Shanghai Suburban Adult Cohort and Biobank (SSACB) study. The baseline investigation started from June 2016 to December 2017. The details of the cohort profile have been well described previously [18]. Participants aged between 20 and 74 years and living for at least 5 years in the district were recruited from four Songjiang communities (Xinqiao, Zhongshan, Maogang, and Sheshan) by stratified multistage random sampling. In this analysis, 36,404 participants completed the physical examinations, questionnaire interviews, and biochemical tests. After excluding the participants with a stroke history before investigation (*n* = 1129); missing data on blood lipids (*n* = 341); and other key variables, such as height, weight, and systolic blood pressure (SBP) (*n* = 640), 34,294 subjects were deemed included in the analysis. The participant inclusion and exclusion criteria are depicted in Fig. 1. All participants signed written informed consent before the research. The cohort protocol was approved by the Ethics Committee on Medical Research at the School of Public Health, Fudan University (IRB#2016-04-0586).

### Questionnaire interview and anthropometric measurement

Information on sociodemographic characteristics (sex, age, education level, and retirement status), self-reported chronic disease history (hypertension, diabetes, and stroke), and lifestyle (smoking, alcohol consumption, and physical activities) was collected by well-trained staff using structured questionnaires through face-to-face interviews. The questionnaire interview was administered on an Android tablet computer with audio recording and paperless data input. Of the recording files, 5% were randomly selected and checked to ensure the quality of the interviews. Anthropometric measurements were conducted with standardized methods by clinicians at the Community Health Service Centers and included weight, height, and blood pressure (BP). BP was measured with a digital sphygmomanometer at least three times after a 5-min rest to document the SBP and diastolic BP (DBP).

### Laboratory assays

Fasting for at least 8 h was required before collecting blood samples in the morning. An automatic biochemical analyzer (Roche Cobas C501) was used to test serum lipids with colorimetry (TG) and enzymatic colorimetry (LDL-C, HDL-C, and TC) methods. A Roche Modular P800 automatic biochemical analyzer was used to test fasting plasma glucose (FPG) with hexokinase methods. A Tosoh G8 automatic glycohemoglobin analyzer was used to measure glycated hemoglobin (HbA1c) with high-pressure liquid chromatography methods. Serum uric acid (SUA) was tested by a Roche Cobas C702 with colorimetry methods. Nontraditional lipids were calculated based on conventional lipids in clinical practice, including non-HDL-C (calculation method: TC minus HDLC) and three lipid ratios: LDL-C/HDL-C, TG/HDL-C, and TC/HDL-C [19].

### Assessment of stroke and follow-up

Follow-up was performed according to the health information system. The outcomes were fatal and nonfatal IS events and were collected based on the Cardiovascular and Cerebrovascular Disease Registration and Reporting System, the electronic medical record (EMR) system, and the cause-of-death surveillance system, which can be matched by a unique identification (ID) number: the ID card number. These online information systems contained a detailed record of the name of a disease diagnosis, date of diagnosis, cause and date of death, and date of onset. The Tenth Revision of the International Classification of Diseases (ICD-10) codes were I63–I64 [20]. Transient ischemic attacks were not included. Participants with self-reported or history of diagnosed stroke events were excluded from the baseline to ensure that the participants had no history of earlier stroke. The earliest onset of IS event or death due to the first IS onset after the baseline was recorded as an outcome. The outcomes were documented by the researchers from the baseline date to December 31, 2021.

### Definition of variables

Hypertension was defined as SBP/DBP ≥ 140/90 mmHg or with a previous diagnosis history [21]. Diabetes was defined as a high level of FPG (≥ 7.0 mmol/L) or HbA1c (≥ 6.5%) or with a previous diagnosis history [22]. The definition of chronic kidney disease (CKD) was persistent abnormal kidney function or kidney impairment, including an estimated glomerular filtration rate (eGFR) < 60 mL/min/1.73 m^2^ or hematuria or proteinuria [23]. Hyperuricemia (HUA) was defined as high levels of SUA (in women, ≥ 360 μmol/L; in men, ≥ 420 μmol/L) [24]. The smoking index was assessed by multiplying packets per day by the smoking years [25]. The definition of current alcohol drinking was drinking ≥3 times per week in the past 6 months. Physical activities were assessed as the metabolic equivalent task (MET) value multiplied by the total number of minutes per week [26] and were divided into three categories: low (MET-mins/week < 2000), moderate (2000 ≤ MET-mins/week < 6000), and high (MET-mins/week ≥6000) [27].

### Statistical analysis

The distribution of data was assessed by the Kolmogorov–Smirnov method. The mean ± standard deviation or median (P25–P75) are described for continuous data. The frequency with corresponding column percentages is presented for categorical data. The baseline characteristics were described according to the participants’ sex. Continuous data were analyzed with Student’s t test (normal distribution) or the Wilcoxon-Mann–Whitney U test (nonnormal distribution). The *χ*^*2*^ test was used to analyze categorical data. The incidence density trend of IS in different lipid quantile groups was tested by the Cochran-Armitage trend test. The relationship between lipids and the risk of IS was studied with Cox regression models. The proportional hazard was tested with the Schoenfeld individual test. The blood lipid levels and lipid ratios were modeled as tertile categories, and the lowest group was considered the reference. Sex, age, retirement status, education level, body mass index (BMI), alcohol consumption, smoking index, physical activities, diabetes, CKD, hypertension, and HUA were fully adjusted in the models. When individual lipids were analyzed, other lipids were adjusted accordingly. Subgroup analysis was conducted by sex, age, and hypertension status. The multiplicative terms of variables were included in the Cox regression models to test the multiplicative interactions. Restricted cubic spline (RCS) analysis with four knots was applied to study the dose–response association between four nontraditional blood lipids and IS. A two-sided *P* value < 0.05 was considered statistically significant. All data were analyzed using SAS 9.4 software (SAS Institute Inc., Cary, NC, USA).

## Results

### Baseline characteristics and outcome data

**Fig. 2:**
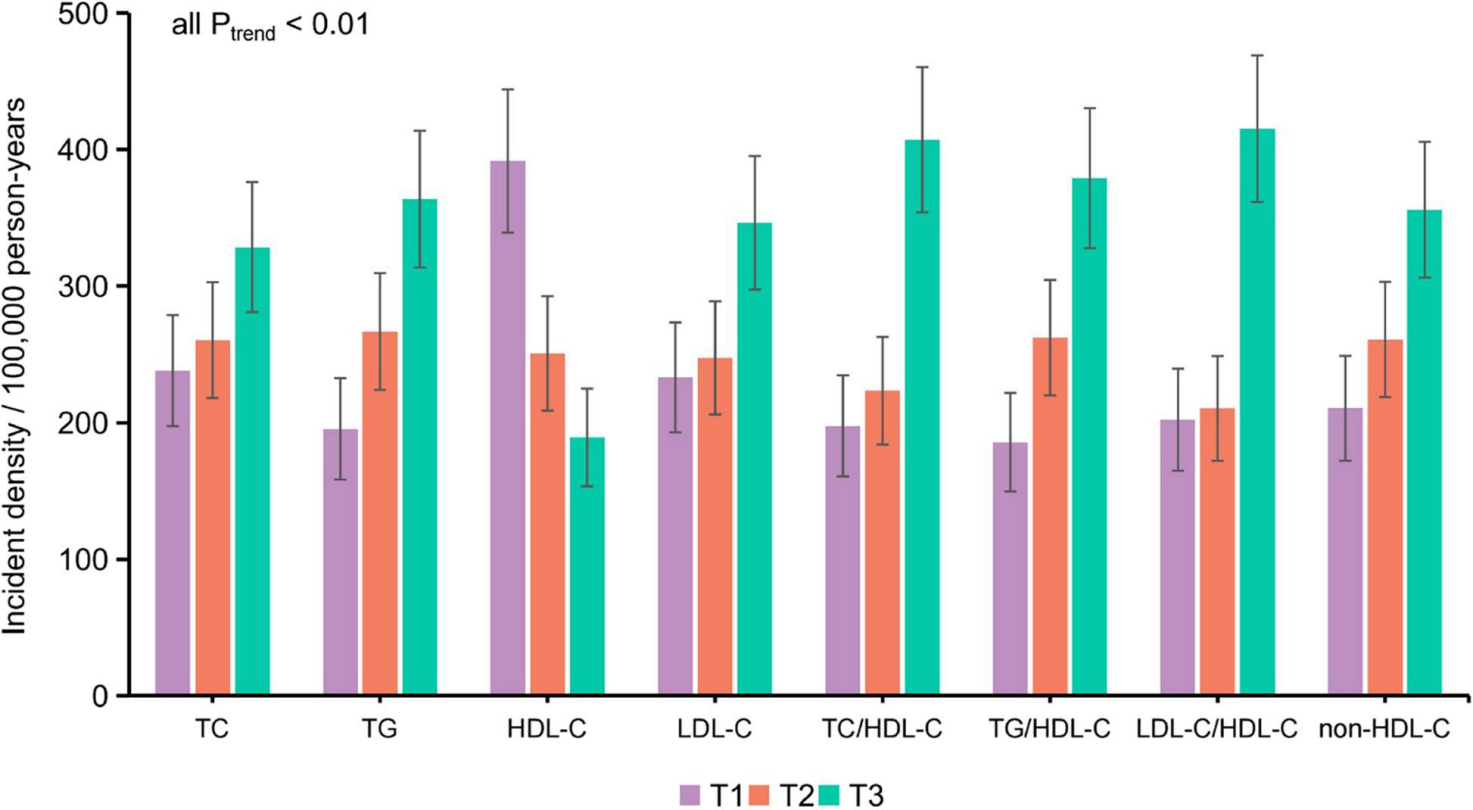
Incidence density of IS according to lipids and lipid ratios by tertile category

The study included 34,294 participants without stroke at baseline. Table 1 demonstrates the participants’ baseline characteristics based on sex. The median age of the participants was 58 (50–65) years. The mean or median nontraditional lipid values at baseline were as follows: TC/HDL-C ratio, 3.71 ± 1.18; TG/HDL-C ratio, 0.96 (0.63–1.55); LDL-C/HDL-C ratio, 2.08 ± 0.79; and non-HDL-C, 3.53 ± 0.95. Male participants were slightly older, had greater diabetes, hypertension, and HUA prevalence, and had higher BMI, TG, and lipid ratio levels than female participants (all *P* <  0.001). A total of 458 incident IS cases were documented after a total of 166,380 person-years of follow-up (median follow-up: 4.97 years). The incidence density and 95% *CI* of IS was 275.27 (250.10–300.45) per 100,000 person-years. Analysis of the incidence density according to blood lipids revealed that the incidence density increased by lipid and lipid ratio tertiles (all *P*_trend_ <  0.01) (Fig. 2).

**Table 1:** Comparison of baseline characteristics of participants according to sex

Characteristics^a^	Male (*N* = 13,844)	Female (*N* = 20,450)	Total (*N* = 34,294)	*P* value
Age (years)	59 (51–66)	57 (50–64)	58 (50–65)	< 0.001
Education level (%)				
≤ 6 education years	891 (6.22)	4010 (19.61)	4871 (14.20)	< 0.001
7 ~ 12 education years	11,658 (84.21)	14,656 (71.67)	26,314 (76.73)	
≥ 13 education years	1325 (9.57)	1784 (8.72)	3109 (9.07)	
Retired (%)				
No	6897 (49.82)	7614 (37.23)	14,511 (42.31)	< 0.001
Yes	6947 (50.18)	12,836 (62.77)	19,783 (57.69)	
Smoking index, packet-year (%)				
None-smoker	5852 (42.27)	20,390 (99.71)	26,242 (76.52)	< 0.001
< 20	2203 (15.91)	38 (0.19)	2241 (6.53)	
20~	3143 (22.70)	17 (0.08)	3160 (9.21)	
40~	2646 (19.11)	5 (0.02)	2651 (7.73)	
Alcohol consumption (%)				
Never	9373 (67.70)	20,299 (99.26)	29,672 (86.52)	< 0.001
Former	346 (2.50)	13 (0.06)	359 (1.05)	
Current	4125 (29.80)	138 (0.67)	4263 (12.43)	
Physical activities (%)				
Low	6554 (47.34)	4039 (19.75)	10,593 (30.89)	< 0.001
Moderate	6599 (47.67)	14,485 (70.83)	21,084 (61.48)	
High	691 (4.99)	1926 (9.42)	2617 (7.63)	
Chronic disease (%)				
Diabetes	2027 (14.64)	2723 (13.32)	4750 (13.85)	< 0.001
Hypertension	7596 (54.87)	10,015 (48.97)	17,611 (51.35)	< 0.001
CKD	929 (6.71)	2660 (13.01)	3589 (10.47)	< 0.001
HUA	2300 (16.61)	1794 (8.77)	4094 (11.94)	< 0.001
BMI (kg/m^2^)	24.71 ± 3.20	24.16 ± 3.42	24.38 ± 3.35	< 0.001
TC (mmol/L)	4.79 ± 0.91	5.04 ± 0.94	4.94 ± 0.94	< 0.001
TG (mmol/L)	1.38 (0.99–2.03)	1.32 (0.97–1.85)	1.34 (0.98–1.92)	< 0.001
HDL-C (mmol/L)	1.30 ± 0.34	1.48 ± 0.35	1.41 ± 0.36	< 0.001
LDL-C (mmol/L)	2.70 ± 0.81	2.83 ± 0.84	2.78 ± 0.83	< 0.001
Non-HDL-C (mmol/L)	3.49 ± 0.93	3.55 ± 0.95	3.53 ± 0.95	< 0.001
TC/HDL-C ratio	3.92 ± 1.29	3.56 ± 1.08	3.71 ± 1.18	< 0.001
TG/HDL-C ratio	1.08 (0.70–1.82)	0.89 (0.60–1.40)	0.96 (0.63–1.55)	< 0.001
LDL-C/HDL-C ratio	2.20 ± 0.84	2.00 ± 0.74	2.08 ± 0.79	< 0.001

^a^Categorical data are expressed as the frequency (%). Continuous data are presented as the median and interquartile range (nonnormally distributed data) or mean and standard deviation (normally distributed data)

### Association of blood lipids with IS

**Fig. 3:**
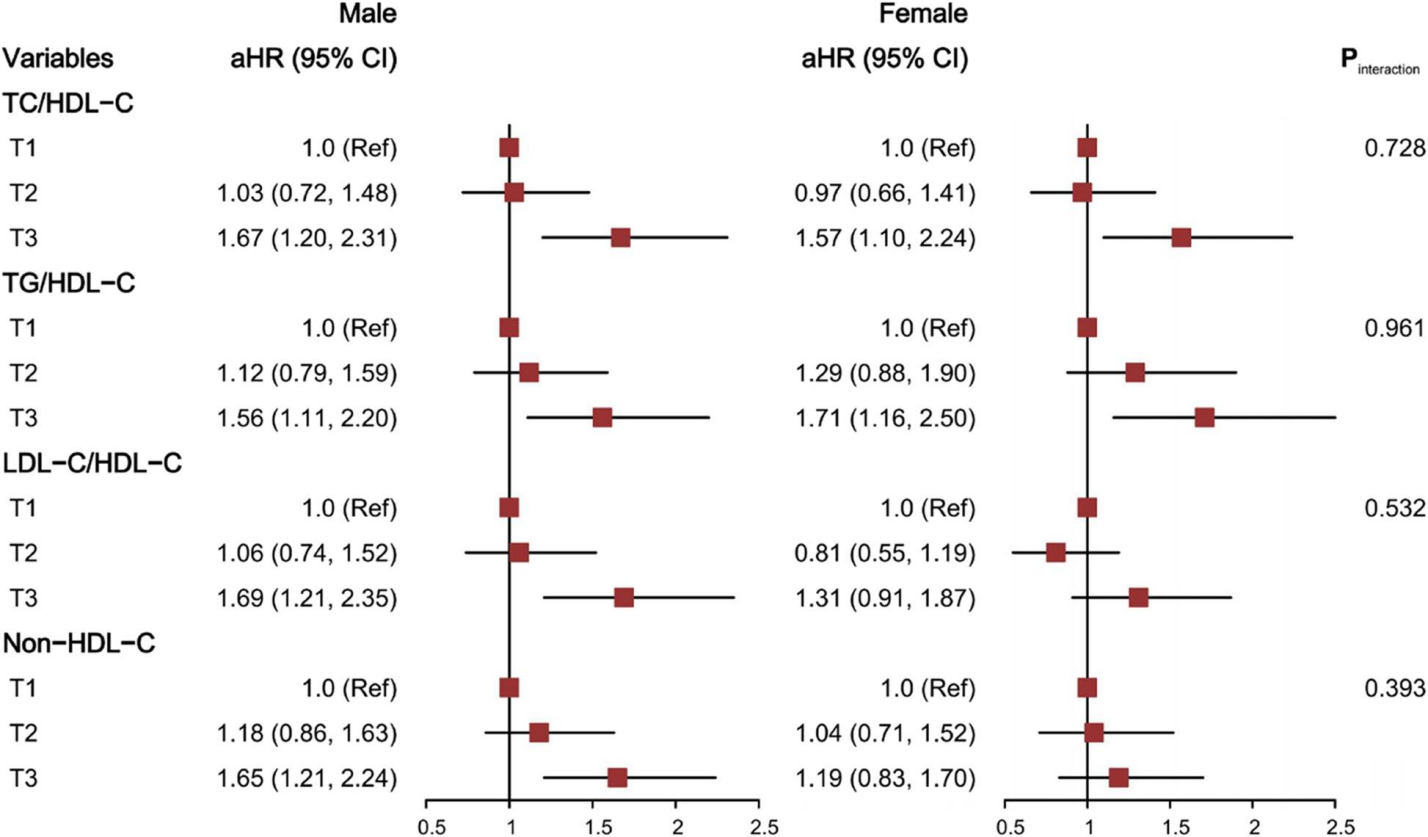
*HRs*^†^ and 95% *CIs* of IS by tertile category of lipid variables according to sex. ^†^Adjusted variables: age, retirement status, education level, BMI, alcohol consumption, smoking index, physical activities, hypertension, diabetes, CKD, and HUA. For TG/HDL-C, TC and LDL-C were additionally adjusted. For LDL-C/HDL-C, TG was additionally adjusted

The Schoenfeld individual test indicated that the risk of lipids on IS had no significant time trends (*P* > 0.05). The association of lipids according to tertile category with IS risk is shown in Table 2. After controlling for potential confounders (age, sex, retirement status, education level, BMI, alcohol consumption, smoking index, physical activities, hypertension, diabetes, CKD, HUA, and other lipids), the highest tertiles were positively related to IS risk when compared with the lowest tertiles, except HDL-C, which had negative associations. The adjusted *HR* increased by blood lipid tertile except for the HDL-C group, which demonstrated a negative trend (all *P*_trend_ <  0.05). The *HRs* and 95% *CIs* of the highest tertiles were 1.63 (1.28–2.07) for TC/HDL-C, 1.65 (1.28–2.13) for TG/HDL-C, 1.51 (1.18–1.92) for LDL-C/HDL-C, and 1.43 (1.13–1.81) for non-HDL-C and showed a greater risk for IS than TC (1.32, 1.03–1.68) and LDL-C (1.36, 1.08–1.71). The analysis was further stratified according to sex (male and female, Fig. 3), age group (< 60 and ≥ 60 years old, Fig. S1), and hypertension status (without and with hypertension, Fig. S2) and determined that the positive association was still statistically significant in males, participants aged ≥60 years, and participants with hypertension. Non-HDL-C and LDL-C/HDL-C presented no significant association with IS risk in the female, < 60 years, and nonhypertension groups. These subgroup variables and lipids demonstrated no interaction effect on IS risk (*P*_interaction_ > 0.05).

**Table 2:** *HRs* and 95% *CIs* of IS by tertile category of lipids

	Cases/N	Ischemic stroke	
		Model 1^a^	Model 2^a^
TC, mmol/L^b^			
T1 (< 4.51)	132/11,358	1.0 (ref)	1.0 (ref)
T2 (4.51–5.24)	144/11,427	1.05 (0.83, 1.32)	1.07 (0.84, 1.36)
T3 (≥ 5.25)	182/11,509	1.31 (1.05, 1.65)	1.32 (1.03, 1.68)
*P*_trend_		0.015	0.022
TG, mmol/L^c^			
T1 (< 1.09)	107/11,206	1.0 (ref)	1.0 (ref)
T2 (1.09–1.66)	149/11,555	1.32 (1.03, 1.69)	1.22 (0.94, 1.57)
T3 (≥ 1.67)	202/11,533	1.87 (1.48, 2.37)	1.58 (1.22, 2.04)
*P*_trend_		< 0.001	< 0.001
HDL-C, mmol/L^c^			
T1 (< 1.25)	213/11,299	1.0 (ref)	1.0 (ref)
T2 (1.25–1.52)	137/11,292	0.62 (0.50, 0.77)	0.64 (0.51, 0.80)
T3 (≥ 1.53)	108/11,703	0.53 (0.42, 0.68)	0.59 (0.46, 0.76)
*P*_trend_		< 0.001	< 0.001
LDL-C, mmol/L^b^			
T1 (< 2.41)	129/11,330	1.0 (ref)	1.0 (ref)
T2 (2.41–3.06)	137/11,410	1.01 (0.79, 1.28)	1.07 (0.84, 1.36)
T3 (≥ 3.07)	192/11,554	1.32 (1.06, 1.66)	1.36 (1.08, 1.71)
*P*_trend_		0.010	0.007
TC/HDL-C			
T1 (< 3.12)	110/11,373	1.0 (ref)	1.0 (ref)
T2 (3.12–3.95)	124/11,446	1.05 (0.82, 1.36)	1.00 (0.77, 1.30)
T3 (≥ 3.96)	224/11,475	1.87 (1.48, 2.35)	1.63 (1.28, 2.07)
*P*_trend_		< 0.001	< 0.001
TG/HDL-C^c^			
T1 (< 0.73)	102/11,228	1.0 (ref)	1.0 (ref)
T2 (0.73–1.29)	147/11,615	1.32 (1.03, 1.70)	1.20 (0.92, 1.55)
T3 (≥ 1.30)	209/11,451	1.98 (1.56, 2.51)	1.65 (1.28, 2.13)
*P*_trend_		< 0.001	< 0.001
LDL-C/HDL-C^d^			
T1 (< 1.70)	113/11,426	1.0 (ref)	1.0 (ref)
T2 (1.70–2.29)	116/11,345	0.99 (0.76, 1.28)	0.93 (0.72, 1.22)
T3 (≥ 2.30)	229/11,523	1.78 (1.42, 2.23)	1.51 (1.18, 1.92)
*P*_trend_		< 0.001	< 0.001
Non-HDL-C, mmol/L			
T1 (< 3.09)	116/11,276	1.0 (ref)	1.0 (ref)
T2 (3.09–3.84)	146/11,548	1.18 (0.92, 1.50)	1.13 (0.89, 1.45)
T3 (≥ 3.85)	196/11,470	1.58 (1.26, 2.00)	1.43 (1.13, 1.81)
*P*_trend_		< 0.001	0.002

^a^Model 1: adjusted variables: age and sex; Model 2: adjusted variables: age, sex, retirement status, education level, BMI, alcohol consumption, smoking index, physical activities, hypertension, diabetes, CKD, and HUA

^b^HDL-C and TG were additionally adjusted

^c^LDL-C and TC were additionally adjusted

^d^TG was additionally adjusted

### Dose–response relationship of nontraditional blood lipids with IS

**Fig. 4:**
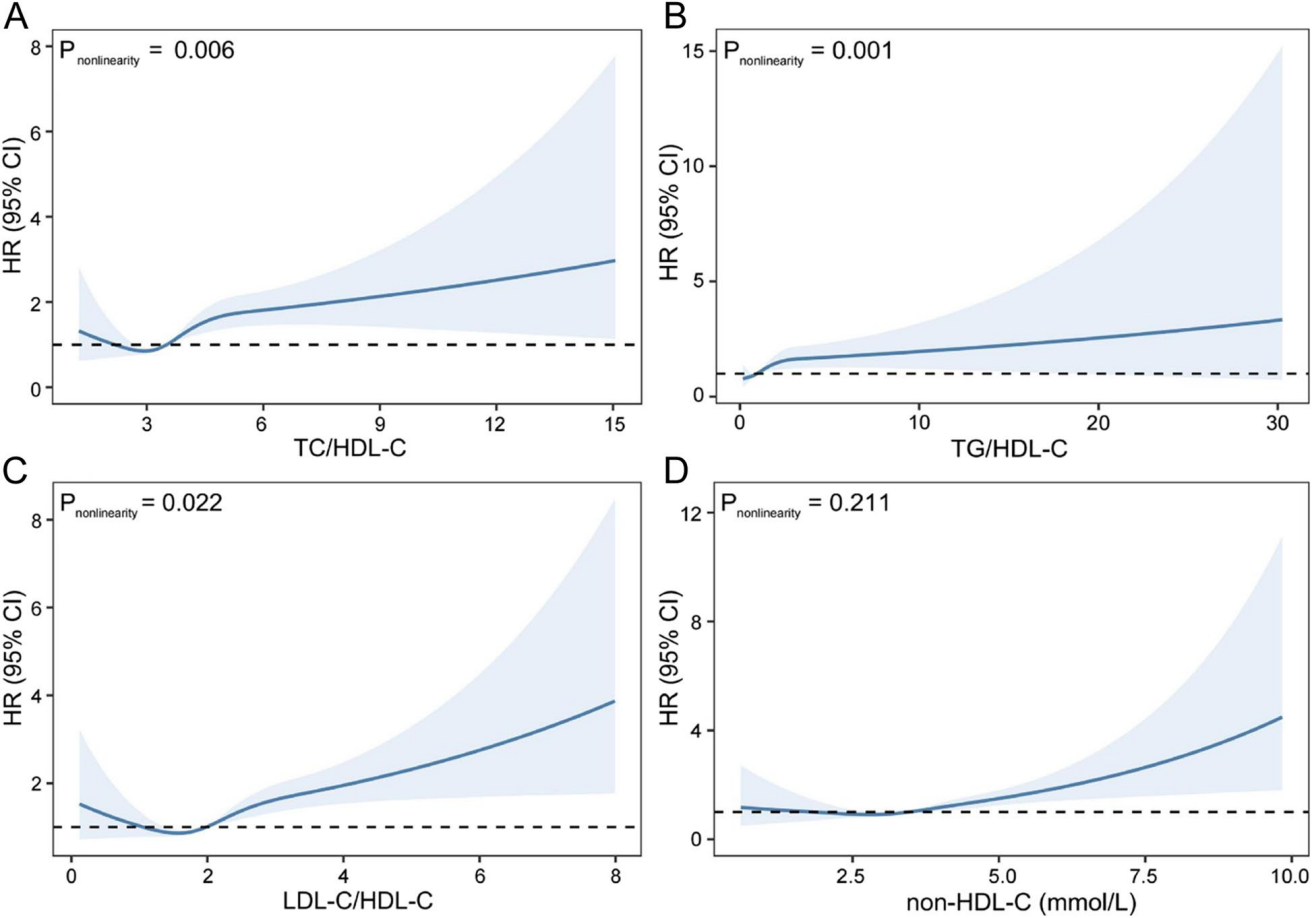
RCS analysis of the relationship between the four nontraditional blood lipid levels and IS (**A**–**D**). Adjusted variables: age, sex, retirement status, education level, BMI, alcohol consumption, smoking index, physical activities, hypertension, diabetes, CKD, and HUA. For TG/HDL-C, TC and LDL-C were additionally adjusted. For LDL-C/HDL-C, TG was additionally adjusted

Nontraditional lipids were studied for their nonlinear connection with IS (Fig. 4). After controlling for confounders, the RCS curves presented a nonlinear relationship with IS (*P*_nonlinearity_ < 0.05) (Fig. 4A–C). When the lipid ratio was > 3.47 (TC/HDL-C), > 0.92 (TG/HDL-C), and > 1.98 (LDL-C/HDL-C), the plot showed increased IS risk. No such nonlinear associations were observed for non-HDL-C (*P*_nonlinearity_ > 0.05), but a positive association between non-HDL-C and IS was observed (Fig. 4D).

## Discussion

The study revealed that the four nontraditional lipids were all positively related to IS risk. The nontraditional lipids demonstrated a stronger association with IS than TC and LDL-C. The subgroup analysis determined a similar positive association for males, older age, and participants with hypertension. Furthermore, the RCS analysis showed a nonlinear relationship between the lipid ratios and IS.

### Comparisons with other studies and what does the current work add to the existing knowledge

Atherosclerosis is a fundamental cause of stroke and heart disease, and an inflammatory response caused by lipoprotein is a critical initiating event that promotes atherosclerosis development [28]. It has been proven that lowering LDL-C reduces CVD risks [29, 30], but a few studies have reported that residual cardiovascular risk remained and that low LDL-C showed greater risks of all-cause mortality and intracerebral hemorrhage [31, 32]. Recently, nontraditional lipids were proposed as valuable predicting factors rather than individual lipids for CVD events [33–36]. Some researchers have reported that lipoprotein ratios reflect metabolic and clinical interactions among lipid components, demonstrating the balance between antiatherogenic and proatherogenic lipoproteins, while an imbalance indicates greater cardiovascular risk [37, 38]. As expected, the findings on the correlation between nontraditional lipids and IS risk are consistent with those of other studies [11–13, 35, 39]. Zheng et al. [11] reported that the four nontraditional blood lipids were related to higher IS risk among hypertensive individuals. Zhou et al. [13] reported a positive association of TG/HDL-C with IS risk among Chinese individuals. The Framingham Study [35] reported that high TC/HDL-C was related to higher IS risk in middle-aged and elderly people (*HR*, 1.47; 95% *CI*, 1.15–1.87). The National Lipid Association recommended non-HDL-C as the major prevention target of ASCVD [40], and Saito et al. [39] concluded that non-HDL-C presented a positive association with large-artery occlusive infarction (*HR*, 2.05; 95% *CI*, 1.07–3.93). Nevertheless, some inconsistency exists. Imamura et al. [41] reported that the association was not statistically significant between non-HDL-C and IS risk (*HR*, 1.01; 95% *CI*, 0.89–1.16). The PRIME Study [14] reported that non-HDL-C, TC/HDL-C, and LDL-C/HDL-C were not robust predictors for IS but were for coronary heart disease only. These inconsistent results may be caused by the differences in study design and method, participants’ characteristics, and the covariates adjusted in the multivariate models.

The subgroup analysis determined that the positive association was statistically significant, especially in the male, older, and hypertension groups. Older age and hypertension are known risk factors for stroke [4], and previous studies reported that the stroke prevalence and incidence were higher in males than females [2, 42], indicating that lowering traditional lipids may be more beneficial for stroke prevention in these high-risk groups. No previous studies explored nonlinear relations between lipid ratios and stroke but only reported a U-shaped association of non-HDL-C with stroke [39]. In the analysis exploring the dose–response relationship, the present study provided additional knowledge that the IS risk increased when the TC/HDL-C, TG-HDL-C, and LDL-C/HDL-C ratios were > 3.47, > 0.92, and > 1.98, respectively. To date, statins are applied in clinical practice to lower cholesterol concentrations to reduce CVD events and are often combined with cholesterol absorption inhibitors (such as ezetimibe) and proprotein convertase subtilisin/kexin type 9 (PCSK9) inhibitors [8, 43, 44]. Nevertheless, the evidence supports the idea that residual inflammatory risk persists when statins are used in combination with PCSK9 inhibitors [44]. The mechanism of the relationship of nontraditional lipid markers with stroke and whether these markers can be used as a new therapeutic target should be confirmed by further studies.

### Strength and limitations

A comprehensive analysis of the relationship between nontraditional lipids and IS was conducted based on a prospective cohort study with strict quality control measures. The study determined the risk threshold of the lipid ratios, as there is currently no acknowledged cutoff for clinical practice. This is the first study to show a nonlinear relationship between nontraditional serum lipids and IS risk in community residents in China. In China, population screening for stroke, which is conducted in high-risk populations, is an essential step in primary prevention [45]. The positive association between nontraditional lipids and stroke and the cutoff found in this study may help determine the high-risk groups for stroke primary prevention and provide a reference for stratifying cardiovascular risk by nontraditional lipid parameters.

However, the study presents several limitations. First, data on antilipemic drug usage were not collected, and dietary conditions were not analyzed, which were possible confounders that may have affected the results. Second, part of the history of earlier strokes was self-reported and may have led to the inclusion of participants with earlier minor strokes. Furthermore, the outcomes were collected based on information systems; inevitably, there will be omissions. However, loss to follow-up was minimized, and new IS events were identified through data linkages across these online information systems. Third, only people from Songjiang district, Shanghai, were included, so the generalization of these results to other populations is limited. Finally, the lipid profile was measured only once at baseline. Therefore, future studies should increase the follow-up duration and increase the number of indicator measurements to better understand the association of serum lipids with stroke, especially nontraditional lipids.

## Conclusion

This study provides evidence that four nontraditional blood lipids are positively associated with IS risk. Maintaining a relatively low lipid ratio can be beneficial for preventing IS occurrence. Therefore, it is recommended that these nontraditional blood lipid parameters be considered in the management of blood lipids in clinical practice. The findings of this study may help provide guidelines for stroke primary prevention and provide a reference for stratifying cardiovascular risk by nontraditional lipid parameters.

## Supplementary Information

**Additional file 1: Fig. S1.** *HR*s and 95% *CIs* of IS by tertile category of lipid variables in different age groups. **Fig. S2.** *HR*s and 95% *CIs* of IS by tertile category of lipid variables in population with or without hypertension.

## Data Availability

The dataset used and analyzed during the current study is available from the corresponding author on reasonable request.

## Abbreviations

HDL-C: High-density lipoprotein cholesterol
TC: Total cholesterol
TG: Triglyceride
LDL-C: Low-density lipoprotein cholesterol
RCS: Restricted cubic spline
IS: Ischemic stroke
HR: Hazard ratio
CI: Confidence interval
CVD: Cardiovascular disease
ASCVD: Atherosclerotic cardiovascular disease
SBP: Systolic blood pressure
DBP: Diastolic blood pressure
BP: Blood pressure
SSACB: Shanghai Suburban Adult Cohort and Biobank
HbA1c: Glycated hemoglobin
FPG: Fasting plasma glucose
SUA: Serum uric acid
HUA: Hyperuricemia
EMR: Electronic medical record
ID: Identification
ICD-10: The Tenth Revision of the International Classification of Diseases
CKD: Chronic kidney disease
eGFR: Estimated glomerular filtration rate
MET: Metabolic equivalent task
BMI: Body mass index
PCSK9: Proprotein convertase subtilisin/kexin type 9
