# Supplementary material for: Association between four nontraditional lipids and ischemic stroke: a cohort study in Shanghai, China
Source: Lipids Health Dis. 2022 Aug 16;21:72. doi: 10.1186/s12944-022-01683-1 (PMC9380319; doi:10.1186/s12944-022-01683-1)
Supplement: Supplementary file 1 — Additional file 1: Fig. S1. HRs and 95% CIs of IS by tertile category of lipid variables in different age groups. Fig. S2. HRs and 95% CIs of IS by tertile category of lipid variables in population with or without hypertension. [file 12944_2022_1683_MOESM1_ESM.docx]

**SUPPLEMENTAL MATERIALS**

**[Contents](javascript:;)**

1. **Fig. S1** *HR*s and 95% *CIs* of IS by tertile category of lipid variables in different age groups
2. **Fig. S2** *HR*s and 95% *CIs* of IS by tertile category of lipid variables in population with or without hypertension


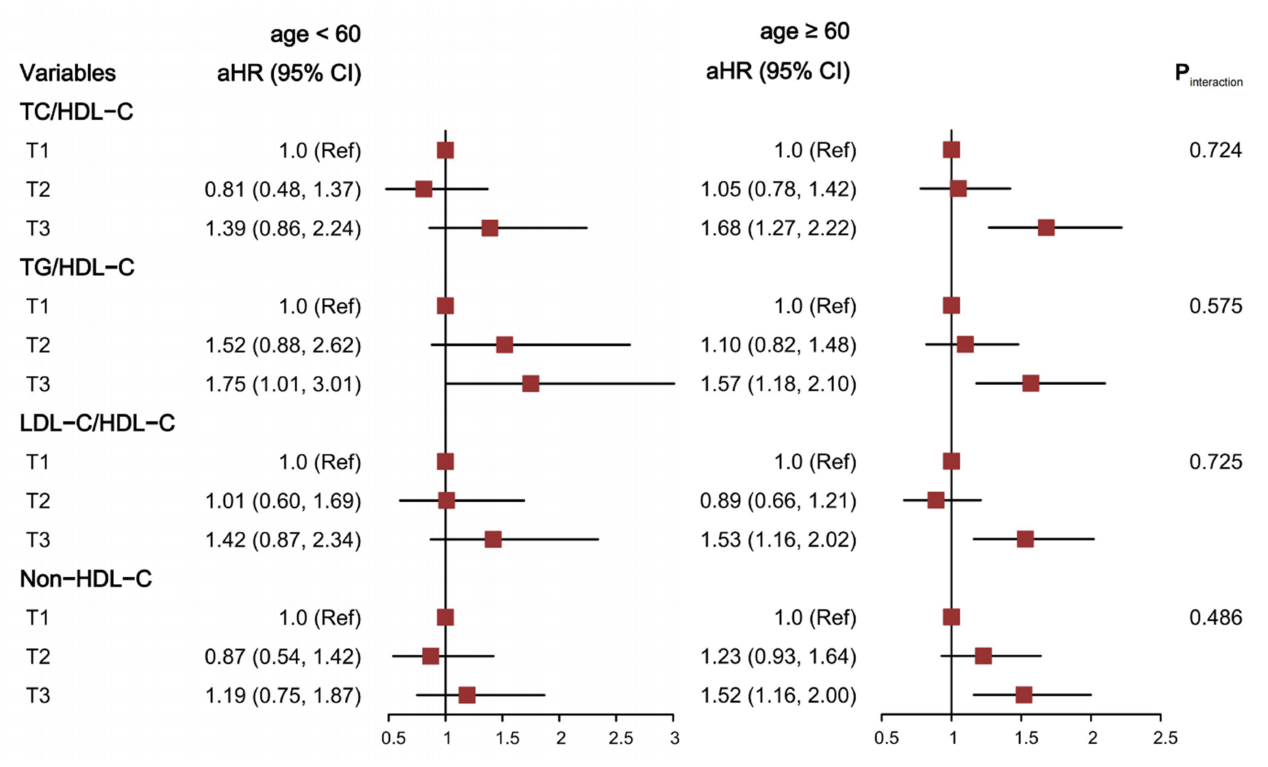


**Figure S1** *HR*s^†^ and 95% *CIs* of IS by tertile category of lipid variables in different age groups. ^†^Adjusted variables: sex, retirement status, education level, BMI, alcohol consumption, smoking index, physical activities, hypertension, diabetes, CKD, and HUA. For TG/HDL-C, TC and LDL-C were additionally adjusted. For LDL-C/HDL-C, TG was additionally adjusted.


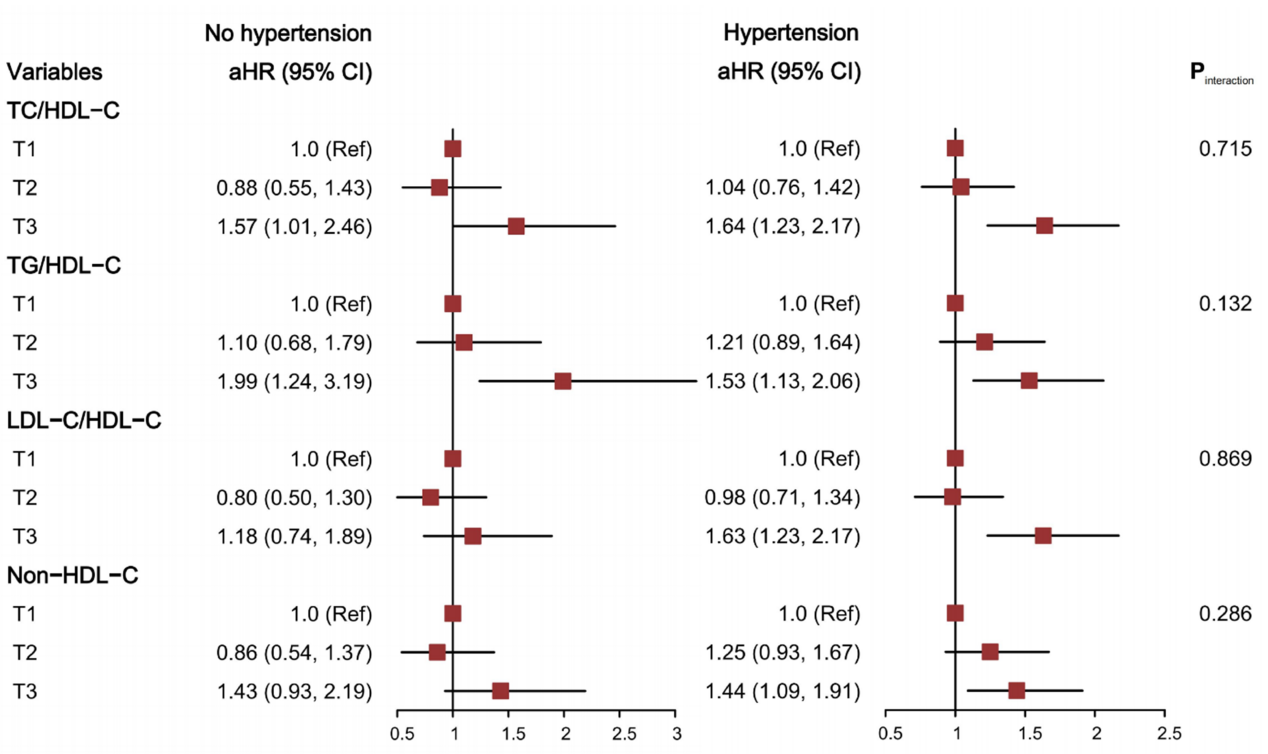


**Figure S2** *HR*s^†^ and 95% *CIs* of IS by tertile category of lipid variables in population with or without hypertension. ^†^Adjusted variables: age, sex, retirement status, education level, BMI, alcohol consumption, smoking index, physical activities, diabetes, CKD, and HUA. For TG/HDL-C, TC and LDL-C were additionally adjusted. For LDL-C/HDL-C, TG was additionally adjusted.
